# Sleep and Socioemotional Outcomes Among Sexual and Gender Minority Adolescents: A Longitudinal Study

**DOI:** 10.1007/s10508-023-02732-1

**Published:** 2023-11-22

**Authors:** Mark Lawrence Wong, Jason M. Nagata, Manuela Barreto

**Affiliations:** 1 Department of Social and Behavioural Sciences, City University of Hong Kong, Tat Chee Avenue, Kowloon, Hong Kong; 2 Division of Adolescent & Young Adult Medicine, Department of Pediatrics, University of California, San Francisco, CA USA; 3 Department of Psychology, University of Exeter, Devon, UK

**Keywords:** Sleep quality, Circadian rhythm, LGBT, Adolescents, Sexual orientation, Gender nonconformity

## Abstract

Sexual and gender minority adolescents face specific stressors associated with their minority status, leading to compromised socioemotional outcomes and sleep. Sleep patterns have, in turn, been found to predict socioemotional outcomes. As such, we aimed to investigate whether healthy sleep patterns prospectively predicted socioemotional outcomes, and whether associations varied as a function of sexual and gender minority status. We conducted a secondary data analysis among adolescents at age 14 to 17, from a nationally representative prospective cohort in the United Kingdom (*N *= 8923, mean age = 13.8), with 7021 cisgender heterosexual, 1801 sexual minority, and 101 gender minority adolescents. Indicators of socioemotional outcomes included self-esteem, emotional well-being, peer relationships, and relationship with parents. The results showed that gender minority adolescents reported the worst sleep patterns and socioemotional outcomes, followed by sexual minority and cisgender heterosexual adolescents. Results from multi-group path analyses showed that emotional well-being, self-esteem, and peer relationships at age 17 were prospectively predicted by sleep patterns and the associations were moderated by sexual and gender minority status. Influence of sleep patterns, particularly sleep initiation problems and frequent awakening, on socioemotional outcomes was greater among gender minority adolescents, when compared to cisgender heterosexual adolescents. In conclusion, positive socioemotional outcomes were predicted by healthy sleep patterns. The potential role of sleep health in optimizing socioemotional outcomes among sexual and gender minority adolescents might warrant further investigation in prevention or intervention studies.

**Supplementary Information:**

The online version contains supplementary material available at 10.1007/s10508-023-02732-1.

## Introduction

Sexual and gender minority adolescents (SGMA) face increased stressors compared to cisgender heterosexual adolescents (Meyer, 2003), which exposes them to an increased risk of poor socioemotional outcomes. This includes poor emotional well-being (Baams et al., 2015), low self-esteem (Bauermeister et al., 2010), peer relationship problems (Bhushan et al., 2019), and poor relationship with parents (Lam et al., 2004). Yet, there is limited understanding about the associated risk and protective factors. In this study, we aimed to investigate the role of a modifiable lifestyle factor, bedtime/sleep quality, in the development of poor socioemotional outcomes among SGMA.

### Socioemotional Outcomes in Sexual and Gender Minority Adolescents

SGMA are susceptible to both general and minority-specific stressors, which increase their vulnerability to poor socioemotional outcomes. Regardless of sexual or gender identity, adolescents experience rapid changes in biological, cognitive and socioemotional aspects during puberty which increase their vulnerability to heightened stress, poor sleep and well-being (Carskadon, 2011; Graber & Archibald, 2001; Martin-Storey et al., 2020). SGMA are, moreover, chronically exposed to minority stressors, such as homophobic attitudes and behaviors (Hatzenbuehler & Pachankis, 2016; Meyer, 2003), which interact with other stressors to negatively affect their well-being. For example, recent studies showed that puberty is particularly stressful for SGMA, since they became more conscious about their minority status and felt less accepted by their peers (e.g., Goldhammer et al., 2019; Reese et al., 2017). Meta-analytical studies showed that being SGMA doubled the chance of having poor socioemotional outcomes (Butler et al., 2020; Liu et al., 2019). Recent studies with nationally and community representative samples showed that SGMA reported lower satisfaction with their self-esteem, emotional well-being, peer problems, relationships with parents and sleep quality, than cisgender heterosexual adolescents (Fedewa & Ahn, 2011; Lam et al., 2004; Liu et al., 2021; Oginni et al., 2019). For instance, identifying as a SGMA was also found to predict lower self-esteem, which mediated the effect of minority stressors on negative emotional well-being (Mereish et al., 2022). Of note, gender minorities have been found to be more vulnerable to minority stressors than cisgender sexual minorities. For instance, compared with cisgender sexual minority youth, gender minorities reported higher rates of victimization in multiple aspects, including physical assault, sexual victimization and school-based bullying (Sterzing et al., 2017). They also received lower social, family and friend support than cisgender sexual minorities (Newcomb et al., 2020). Results from a national longitudinal cohort study among sexual and gender minority adults in the US (*n* = 5299) also showed gender minorities had a higher prevalence rate of internalized stigma, prejudice or victimization due to sexual orientation, gender identity or gender expression, when compared to cisgender sexual minorities (Flentje et al., 2022). In sum, SGMA, particularly gender minorities, tend to experience minority stressors which are associated with unsatisfactory socioemotional outcomes, making it important to examine whether gender minorities were more vulnerable to poor socioemotional outcomes, as well as the factors that can be amenable to intervention to improve their socioemotional development.

### Sleep and Socioemotional Outcomes

Sleep health has been noted to deteriorate during adolescence, due to a mismatch between adolescents’ delayed bedtime/evening-preference, with early school start time (Carskadon, 2011). Recent studies showed that short sleep duration, or poor sleep quality, are early indicators of later social relationship functioning and emotional well-being (e.g., Huang et al., 2018; Wong et al., 2013). For instance, short sleep duration and poor sleep quality were found to prospectively predict the later development of poorer self-esteem and emotional well-being after adjusting for the corresponding factors at baseline (Wong et al., 2013). Also, a recent longitudinal study found a bidirectional relationship between adolescents’ sleep quality and low levels of peer relationships (Tu & Cai, 2020). Among a nationally representative US sample, adolescents with insufficient sleep (< 7 h) reported having poorer relationships with their mother and father than their counterparts with sufficient sleep (7–10 h) (Mueller et al., 2011). A recent 14-day diary study showed that poor sleep quality could predict next-day self-reported provision of support towards a partner and perceived support received from a partner, with such associations mediated by negative affect (Sell et al., 2023). It was concluded that sleep could influence the social relationship process, including the provision and perceived reception of support, partially through sleep’s influence on negative affect. Neuroscience literature also suggested that insufficient sleep was associated with suboptimal functioning of neural circuitry subserving social and emotional functioning, such as empathy (Guadagni et al., 2018), recognizing social cues of affiliative versus threatening behaviors (Goldstein-Piekarski et al., 2015), theory of mind, and social withdrawal (Ben-Simon & Walker, 2018). In relation to sleep quality, subjective sleep complaints have been indicated in a range of mental disorders (e.g., major depressive disorders, posttraumatic disorders), and sleep problems have also been considered as a transdiagnostic behavioral manifestation of underlying cognitive and neurobiological disturbances (Harvey et al., 2011). While bedtime, sleep initiation, and sleep maintenance problems, were suggested to be generally modifiable through short-term treatment (Espie, 2009), their roles in the development of socioemotional outcomes in SGMA have rarely been studied (Martin-Storey et al., 2020).

Butler et al. (2020) suggested that despite the consistent evidence for sleep disparities as a function of sexual and gender identity, as well as for the role of sleep problems as a prospective predictor of poor health outcomes in the general population, the investigation of the contribution of sleep towards socioemotional outcomes in SGMA was still at the beginning stage. They also stated that it is important to examine sleep health among both sexual and gender minorities. Indeed, some sleep problems (e.g., subjective sleep quality) and socioemotional outcomes (e.g., negative mood) were more prevalent among sexual and gender minority individuals than among cisgender heterosexual individuals (Huang et al., 2018; Tabler et al., 2019). One explanation was that, as minority individuals, SGMA face chronic stress, which could disrupt many regulatory systems in our body, including sleep and circadian rhythm. In a recent community survey of 401 lesbian, gay, and bisexual adults, Chan and Fung (2021) found that anticipated discrimination was correlated with greater sleep disturbance, and greater sleep disturbance mediated the association between anticipated discrimination and mental health. It was concluded that anticipated discrimination might lead to the feelings of threat, which heighten an individual’s arousal system and affect sleep quality. Poor sleep quality, in turn, potentially translated the impact of discrimination and perceived threat into poor health outcomes. Yet, the temporal association among minority stressors, sleep and mental health outcomes among sexual minorities are yet to be verified with longitudinal data.

### The Current Study

The primary aim of this study is to identify the role of sleep patterns in socioemotional outcomes among SGMA. For socioemotional outcomes, we are particularly interested in areas encompassing adolescent gender development, such as peer and family relationships, which were relevant to (non)-compliance with gender norms and minority stressors (e.g., Cook et al., 2019), as well as self-esteem, and emotional well-being, which could represent adolescents’ adjustment with the developmental changes. Based on the evidence demonstrating that (1) sleep predicted socioemotional outcomes (e.g., Lam et al., 2004; Wong et al., 2013), (2) that there was a heightened rate of sleep and socioemotional problems among SGMA (e.g., Oginni et al., 2019; Tabler et al., 2019), and that (3) sleep disturbance mediated the effect of some minority stressors (e.g., discrimination) on mental well-being (e.g., Chan & Fung, 2020), we hypothesized that sleep patterns would prospectively predict socioemotional outcomes. Given that gender minorities (GM) have been reported to face a higher level of sleep disturbances and minority stress than sexual minority (SM), and cisgender heterosexual individuals (Butler et al., 2020; Flentje et al., 2022; Newcomb et al., 2020), we hypothesized the effect of sleep disturbance on socioemotional outcomes would be stronger among GM than SM and cisgender heterosexual adolescents. To address these research aims, we conducted secondary data analyses using a nationally representative prospective birth cohort—the Millennium Cohort Study (MCS), which includes more than 10,000 participants born in the four countries of the United Kingdom (Centre for Longitudinal Studies CLS, 2017). The current study’s findings could potentially indicate if healthy sleep patterns can be targeted in socioemotional well-being promotion among SGMA.

## Method

### Participants

In the MCS, the inclusion criteria were infants born between 2000 and 2002 who were identified through the UK government child benefit records. It is a nationally representative cohort with children living in disadvantaged areas, or from ethnic minority backgrounds oversampled to ensure disadvantaged groups were represented in the cohort. The cohort has 18,826 children at baseline (sampled at 9-month-old), and full details of the cohort are elsewhere described (Connelly & Platt, 2014). In this study, we extracted the data from the age-14 and age-17 time-points. The inclusion criteria were: No missing data or invalid responses, e.g., “Don’t know,” “Prefer not to say” or “not applicable,” on the measures of sexual and gender identity (see Measures section for details).

### Procedure

Participants and their parents could choose to complete the questionnaires themselves or have the interviewers administer the questionnaires. In this study, we extracted data on participants’ sexual and gender identity, sleep patterns, socioemotional outcomes, and demographic information.

### Measures

#### Demographic Information

Participants were asked about their age, sex, and ethnicity. The parents of the participants also reported the participants’ assigned sex at birth and household weekly income.

#### Sexual and Gender Minority Status

Apart from assigned sex at birth, two multiple-choice items were administered at age 17 to assess SGMA status. Consistent with published guidelines, (e.g., National Academies of Sciences, Engineering, and Medicine, 2022), GM status was assessed by the participant’s assigned sex at birth and their self-reported gender identity at the time of interview. Participants were asked to indicate which of the following options best described how they currently thought of themselves: “Male,” “Female,” “In any other way, please write in the box” which allowed participants to indicate any other responses (e.g., non-binary, gender fluid). Similar to existing studies (e.g., Mitchell et al., 2022), participants who selected an option different from their assigned sex at birth were categorized as GM. Second, sexual minority status was inferred from individuals’ self-reported sexual identity (National Academies of Sciences, Engineering, and Medicine, 2022), where participants were asked to select the option that best described how they currently thought about themselves, with options including “Completely heterosexual/straight,” “Mainly heterosexual/straight,” “Bisexual,” “Mainly gay or lesbian,” “Completely gay or lesbian,” and “Other.” Participants were categorized into the SM group if they were not a GM and selected an option other than “Completely heterosexual/straight.” Participants were categorized into the cisgender heterosexual group if they were not a GM and selected the option “Completely heterosexual/straight.” For both items, participants could report “Don’t know,” “Prefer not to say” or “Not applicable,” and those who selected these options were not included in the final sample as there was insufficient information to infer their sexual and gender identity.

#### Sleep Patterns

Similar to existing measures of sleep pattern (e.g., Monk et al., 2003), participants’ bedtime and waketime were assessed by self-reported time to go to bed, and to wake up, on a school or no-school night/day separately, at age 14. Sleep initiation problem was assessed by the time taken to fall asleep, and sleep maintenance problem was assessed by the frequency of night with awakening during sleep. While (1) published guidelines suggested that adolescents typically need at least 7–8 h of sleep (Hirshkowitz et al., 2015), (2) adolescents in UK schools tend to start between 8 and 9 am, and (3) traveling time to school can take up to an hour (Illingworth et al., 2019), we considered bedtime later than 11 pm during school nights as problematic, and participants were categorized into either late bedtime or not on this basis. We considered individuals to have a significant sleep initiation problem if they took > 30 min to fall asleep and with frequent awakening if they indicated being awake during the night “a good bit of time, most or all of the time” based on existing studies (e.g., Vallières et al., 2005).

#### Socioemotional Outcomes

The following indicators of socioemotional outcomes were measured at both age 14 and age 17, and their psychometric properties are reported in Table 1. Self-esteem was measured by the Bachman (1970) revision of the Rosenberg (1965) Self-Esteem questionnaire, where participants responded to statements that described the positive evaluation of themselves, e.g., “On the whole, I am satisfied with myself.” Emotional well-being and peer relationships were each measured by the emotion or peer relationship subscales of the Strengths and Difficulties Questionnaire, SDQ (Goodman, 2001). Participants (age 17) or their parents (age 14) responded to statements regarding emotional well-being, e.g., “Often unhappy, down-hearted or tearful” or peer relationships, e.g., “Generally liked by other children.” Relationships with parents were measured with two items, which asked how close participants were to their mother or father. Participants could also indicate if they did not have a mother/father, or were not in contact with them, in which cases their responses were not used in analyzing the association between sleep and relationship with that corresponding parent.

**Table 1:** Between-group comparisons of socioemotional outcomes

	Cronbach’s alpha	Combined	Cisgender Heterosexual	Sexual minority	Gender minority
*Self-esteem *(5–20)					
Age 14	0.91	15.60 (2.9)	15.85 (2.8) ^a^	14.70 (3.2) ^b^	14.07 (3.6) ^b^
Age 17	0.91	15.03 (3.2)	15.35 (3.0) ^a^	13.95 (3.3) ^b^	12.31 (3.4) ^c^
*Emotional Well-being* (0–10)					
Age 14	0.73	8.03 (2.1)	8.10 (2.1) ^a^	7.78 (2.2) ^b^	6.98 (2.5) ^c^
Age 17	0.74	6.49 (2.5)	6.84 (2.3) ^a^	5.27 (2.5) ^b^	4.29 (2.5) ^c^
*Peer Relationships* (0–10)					
Age 14	0.62	8.33 (1.8)	8.42 (1.7) ^a^	8.01 (1.9) ^b^	7.27 (2.1) ^c^
Age 17	0.56	7.88 (1.7)	8.04 (1.6) ^a^	7.33 (1.8) ^b^	6.48 (1.9) ^c^
*Relationship with Mother* (1–4)					
Age 14		3.27 (0.80)	3.30 (0.79) ^a^	3.16 (0.84) ^b^	3.10 (0.89) ^ab^
Age 17		3.23 (0.78)	3.28 (0.76) ^a^	3.09 (0.83) ^b^	2.98 (0.89) ^b^
*Relationship with Father* (1–4)					
Age 14		3.05 (1.0)	3.08 (1.0) ^a^	2.95 (1.1) ^b^	2.88 (1.2) ^ab^
Age 17		2.99 (0.87)	3.05 (0.86) ^a^	2.78 (0.89) ^b^	2.48 (0.89) ^c^

Group comparisons were conducted with family income and ethnicity as covariates; group(s) with different superscript indicate significant group difference after Bonferroni adjustment;

### Statistical Analysis Plan

For the core hypotheses pertaining to the role of sleep in socioemotional outcomes, we conducted a multi-group path analysis with age-14 sleep patterns as the predictor, age-17 socioemotional indicators as the outcomes, and SGMA status as the grouping variable. The age-14 socioemotional indicators, as well as demographic factors were entered as covariates, given they were found to correlate with sleep patterns (e.g., Olds et al., 2010; Guglielmo et al., 2018). Several model fit indices, including a comparative fit index, CFI > 0.95, a root mean square error of approximation, RMSEA < 0.06 with *p *value > 0.05, and standardized root mean square residual, SRMR < 0.80, indicated a good fit between the hypothesized model with the data (Hu & Bentler, 1999). The multi-group analysis was conducted using the unconstrained-constrained approach. The unconstrained model was compared with the fully constrained model and a significant chi-square statistic, *p* < .05, indicated the model-based moderation effect of SM and GM status. A follow-up path-by-path analysis was conducted to assess if the difference on each path across the three groups reached statistical significance, where a critical ratio for differences (CRD) ≥ 1.965 indicated a statistically significant difference. Jamovi, version 2.3, was for all statistical analyses (The Jamovi Project, 2023).

## Results

### Sample Characteristics

In the MCS, 11,872 adolescents participated in the 6th time-point, and 10,757 continued in the 7th time-point, of which 8923 (83.0%) were included in this study. Detailed demographic information is presented in Table 2. Participants were generally aged 13–15 with 7021 (79%) identified as cisgender heterosexual, 1801 (20%) as SM, and 101 (1%) as GM. There was a significantly lower proportion of Black/African/Caribbean and other ethnic groups among GM than the other two groups, χ^2^(10) = 116.39, *p* < .001. The groups also significantly differed in weekly income, *F*(2 8910) = 34.42, *p* < .001, where SM were found to have a significantly higher weekly income than the cisgender heterosexual adolescents, mean difference = 38.98, *p*_bonferroni_ < .001 (Table 2).

**Table 2:** Descriptive information

	Combined		Cisgender heterosexual	Sexual minority	Gender minority
N	8923		7021	1801	101
Age	13.8 (0.45)		13.8 (0.46)	13.8 (0.45)	13.7 (0.47)
Sex – Female/Male	4593/4330		3320/3701^a^	1198/603^b^	75/26^c^
Family weekly income £	426.4 (178.6)		418.5 (176.9) ^a^	457.4 (183.1) ^b^	420.1 (155.2) ^ab^
*Ethnicity*					
White	80.0%		78.2%	86.6%	90.1%
Mixed/multi-ethnic	4.6%		4.4%	5.4%	4.0%
Asian	10.0%		11.5%	4.5%	6.0%
Black/African/Caribbean	3.1%		3.5% ^a^	1.9% ^a^	0% ^b^
Other	2.3%		2.5% ^a^	1.7% ^a^	0% ^b^
*Sleep time *					
Bedtime > 11pm	25.3%		23.1% ^a^	33.2% ^b^	36.6% ^b^
Waketime > 8am	3.4%		3.4%	3.3%	3.0%
*Sleep quality *					
Sleep initiation > 30 min	17.6%		16.3% ^a^	21.7% ^b^	36.6% ^**c**^
Frequent awakening	18.8%		17.9% ^a^	21.7% ^b^	31.7% ^**c**^

Chi-square test was conducted on ethnicity, sleep time and sleep quality measures while ANOVA was used for the remaining variables; group(s) with different superscript indicate significant group difference

### Differences Between Groups in Sleep Patterns and Socioemotional Outcomes

For sleep patterns, there was a significantly higher proportion of individuals with late bedtime among the SM and GM than cisgender heterosexual adolescents, χ^2^(2) = 82.52, *p* < .001. The proportion of individuals with sleep initiation problems, χ^2^(2) = 53.96, *p* < .001, and frequent nocturnal awakening, χ^2^(2) = 24.71, *p* < .001, was highest among GM, followed by SM, and then the cisgender heterosexual adolescents **(**Table 2**)**. For socioemotional outcomes, after controlling for ethnicity and family income, the groups were significantly different on all measures (Table 1). In general, the worst rating on the socioemotional outcomes were found among GM, followed by SM and then cisgender heterosexual adolescents (Supplementary results).

### Prospective Associations Between Sleep and Socioemotional Outcomes Across Sexual and Gender Minority Adolescents

**Fig. 1:**
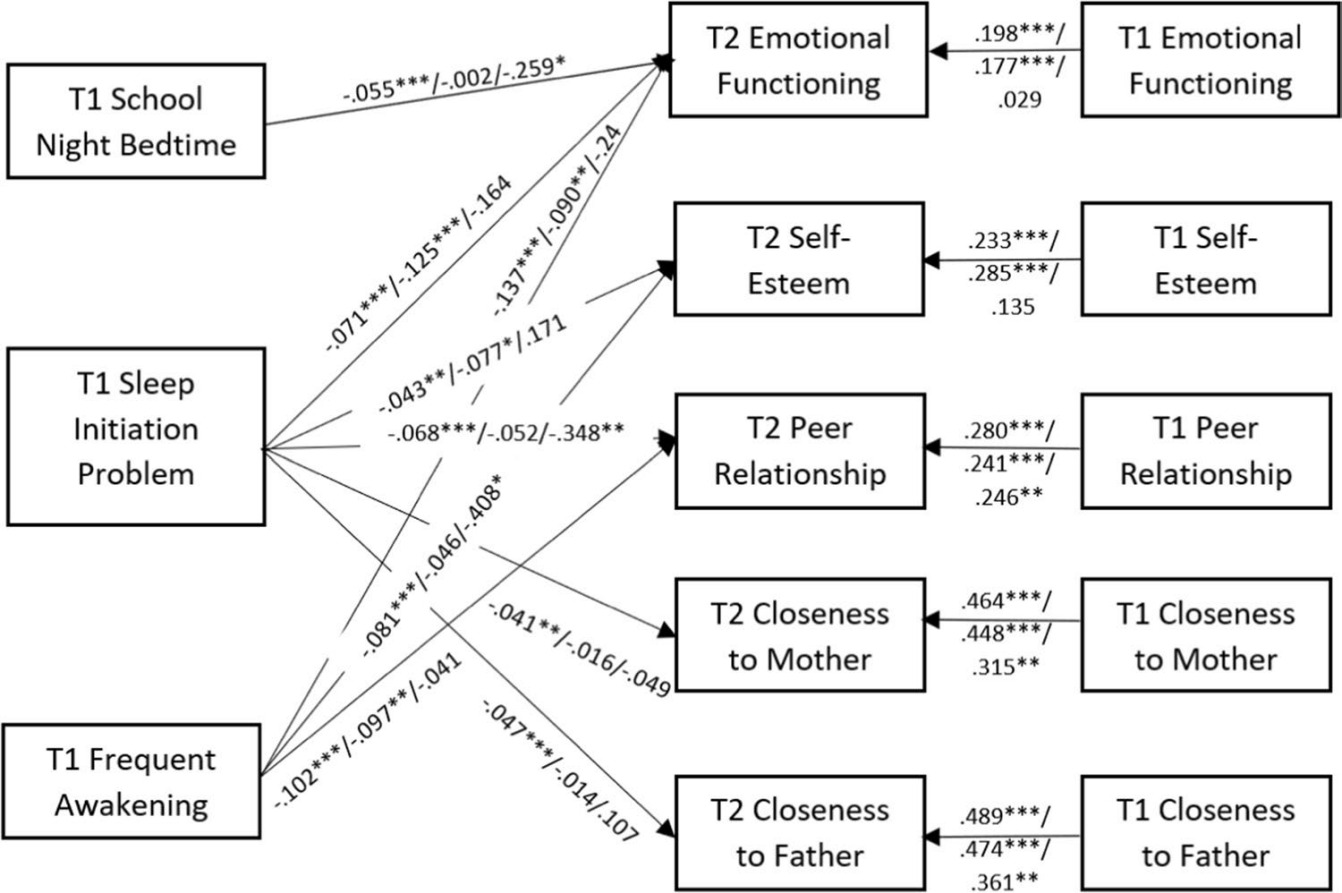
Significant effects of sleep on socioemotional outcomes among cisgender heterosexual, SM and GM adolescents

Results from the unconstrained model achieved good fit indices, CFI = 0.968, RMSEA = 0.054, *p* = .128, SRMR = 0.024, indicating the hypothesized model had a good fit with the data. The non-significant chi-square results, Δχ^2^ (80) = 86.40, *p* = .293, supported measurement invariance across the groups. We then proceed to test a fully constrained model, where the regression coefficients, residual variance and covariance were constrained. The fully constrained model also achieved good fit indices, CFI = 0.953, RMSEA = 0.038, *p* = 1.000, SRMR = 0.027. Results from the chi-square test showed that the two models were significantly different, Δχ^2^ (90) = 169.10, *p <* .001, which indicate significant multi-group moderation. Details of all the significant relationships in the path analysis were summarized in Table 3, Supplementary Tables 1, Fig. 1, and Supplementary Results. The intercorrelation of all study variables could be found in Supplementary Table 2.

**Table 3:** Multi-group path analysis of sleep patterns effects on socioemotional outcomes by sexual and gender minority status

	Cisgender heterosexual		Cisgender sexual minority		Gender minority	
	Est (SE)	Beta	Est (SE)	Beta	Est (SE)	Beta
*Outcome: Emotional Well-being*						
Bedtime	− 0.142 (0.04)***	− 0.055 ^**a**^	− 0.006 (0.08)	− 0.002 ^**b**^	− 0.617( 0.30)*	− 0.259 ^**a**^
Sleep initiation P	− 0.134 (0.03)***	− 0.072 ^**a**^	− 0.234 (0.06)***	− 0.125 ^**b**^	− 0.295 (0.25)	− 0.164 ^**ab**^
Freq awakening	− 0.245 (0.03)***	0.137 ^**a**^	− 0.168 (0.06)**	0.090 ^**b**^	− 0.414 (0.22)	0.244 ^**ab**^
*Outcome: Self-esteem*						
Bedtime	− 0.057 (0.05)	− 0.017	− 0.087 (0.10)	− 0.027	− 0.467 (0.46)	− 0.136
Sleep initiation P	− 0.105 (0.04)**	− 0.043 ^**a**^	− 0.184 (0.08)*	− 0.077 ^**b**^	0.445 (0.36)	.171^**a**^
Freq awakening	− 0.193 (0.04)***	− 0.081 ^**a**^	− 0.111 (0.08)	− 0.046 ^**b**^	-1.00 (0.32)**	− 0.408 ^**c**^
*Outcome: Peer Relationship*						
Bedtime	− 0.021 (0.03)	− 0.012	0.012 (0.06)	0.006	− 0.146 (0.24)	− 0.076
Sleep initiation P	− 0.087 (0.02)***	− 0.068 ^**a**^	− 0.071 (0.04)	− 0.052 ^**a**^	− 0.508 (0.19)**	− 0.348 ^**b**^
Freq awakening	− 0.127 (0.02)***	− 0.103	− 0.132 (0.04)**	− 0.097	− 0.057 (0.17)	− 0.042
*Outcome: Relationship with Mother*						
Bedtime	− 0.007 (0.01)	0.008	0.038 (0.03)	0.045	− 0.115 (0.11)	− 0.146
Sleep initiation P	− 0.025 (0.01)**	− 0.041	− 0.010 (0.02)	− 0.016	− 0.029 (0.09)	− 0.049
Freq awakening	0.005 (0.01)	− 0.009	0.018 (0.02)	0.029	0.023 (0.08)	0.041
*Outcome: Relationship with Father*						
Bedtime	− 0.026 (0.01)	− 0.027	− 0.008 (0.03)	− 0.009	0.316 (0.10)	0.107
Sleep initiation P	− 0.033 (0.01)***	− 0.047	− 0.010( 0.02)	− 0.014	0.090 (0.12)	0.107
Freq awakening	0.002 (0.01)	0.003	0.006 (0.02)	0.009	0.066 (0.09)	0.104

Group(s) with different superscript indicate significant group difference; Sleep initiation P, Sleep initiation problem; Freq awakening, Frequent awakening during sleep; Est, Unstandardized regression coefficient; SE, Standard error; Beta, Standardized regression coefficient; *p < .05, **p < .01, ***p < .001

We proceeded to conduct path-by-path analyses and the details of the results were presented in Table 3. For instance, sleep initiation problems significantly predicted emotional well-being, CRD = − 3.13, and self-esteem, CRD = − 2.11, only in SM and cisgender heterosexual adolescents. In addition, each unit decrease in sleep initiation problems corresponded to a larger increase in emotional well-being and self-esteem among SM, when compared to the cisgender heterosexual adolescents. Also, the effect of sleep initiation problems on peer relationships was only significant in the cisgender heterosexual and GM groups, where each unit decrease in sleep initiation problems corresponded to a larger increase in peer relationships among GM than the cisgender heterosexual adolescents, CRD = − 2.45.

Regarding frequent awakening, its effects on emotional well-being were only significant for the cisgender heterosexual and SM respondents, with each unit decrease in frequent awakening corresponding to a larger increase in emotional well-being among the cisgender heterosexual participants than SM, CRD = − 2.39. The effect of frequent awakening on self-esteem also varied across groups and was only significant among the cisgender heterosexual and GM adolescents, with each unit decrease in nocturnal awakening corresponding to a larger increase in self-esteem among GM, when compared to the cisgender heterosexual adolescents, CRD = 3.67 (Table 3).

## Discussion

This study aimed to assess the role of sleep in socioemotional outcomes among SGMA. We found poorer socioemotional outcomes among SGMA than cisgender heterosexual adolescents, with GM reporting the lowest satisfaction with socioemotional outcomes. After adjusting for demographic and baseline measures of socioemotional outcomes, late bedtime, sleep initiation problems, and frequent awakening prospectively predicted socioemotional outcomes. Of note, sleep’s impact on socioemotional outcomes varied as a function of SGMA status and the implications are discussed in the following sections.

### Sleep and Socioemotional Outcomes Among Sexual and Gender Minority Adolescents

Consistent with our hypothesis, SGMA reported poorer sleep and socioemotional outcomes than cisgender heterosexual adolescents. These results were in line with existing studies, which showed that GM, were exposed to more stressors than SM or cisgender heterosexual adolescents, which potentially leads to poorer socioemotional outcomes (Flentje et al., 2022; Mitchell et al., 2022; Tabler et al., 2019) or sleep health (Levenson et al., 2021; Martin-Storey et al., 2020). Our analyses additionally showed that the disparity in socioemotional outcomes appeared to increase from age 14 to age 17. For instance, GM reported worse self-esteem and relationships with their fathers than SM participants at age 17, but not at age 14. The poorer socioemotional outcomes of GM at age 17 potentially reflected increasing distress with pubertal development, e.g., bodily changes and gender identity, which negatively affected their socioemotional outcomes (Butler et al., 2020; Goldhammer et al., 2019; Hatzenbuehler & Pachankis, 2016). Therefore, our findings suggested that there were significant changes in socioemotional outcomes during this developmental period, which should be considered in prevention and intervention campaigns. While this study was conducted among adolescents at age 14 and age 17, future studies conducted with older youths could further assess if GM consistently experience worse sleep and socioemotional outcomes than other groups.

### The Protective Role of Healthy Sleep Patterns in Socioemotional Outcomes

Results from the multi-group path analyses suggested that, after adjusting for demographic factors and baseline socioemotional outcomes, healthy sleep patterns at age 14 prospectively predicted better socioemotional outcomes at age 17, and the effects varied as a function of SGMA status. Consistent with existing studies conducted among the general youth population (e.g., Wong et al., 2013), for cisgender heterosexual participants, sleep patterns predicted most socioemotional outcomes examined. For instance, better relationships with parents were predicted by less severe sleep initiation problems among cisgender heterosexual adolescents. The relationship between sleep and socioemotional outcomes can be understood based on sleep’s essential role in the brain mechanisms that regulate emotion (Goldstein & Walker, 2014), and cognition (Wong et al., 2020). Alternatively, sleep problems have been considered as a transdiagnostic marker representing diverse underlying dysfunctional behavioral and cognitive processes (Harvey et al., 2011). The absence of sleep problems might therefore also represent good behavioral and cognitive functioning in general.

Among SGMA, to the best of our knowledge, no studies had looked into the prospective association between sleep and socioemotional outcomes. Our results showed that sleep quality measures, significantly predicted emotional well-being, self-esteem, and peer relationships. The magnitude of influence of sleep over these socioemotional outcomes was found to differ across the studied groups, with each unit decrease in sleep initiation problems and frequent awakening corresponding to a larger increase in peer relationships and self-esteem, respectively, among GM when compared to the cisgender heterosexual adolescents. The difference between SM and cisgender heterosexual adolescents was less clear. While the influence of sleep initiation problems on emotional well-being and self-esteem was found to be greater for the SM than for the cisgender heterosexual adolescents, bedtime’s influence on emotional well-being was greater for the cisgender heterosexual adolescents instead. While these patterns of results appear to indicate sleep quality, especially sleep initiation problems, influenced the socioemotional outcomes of SGMA to a larger extent when compared to the cisgender heterosexual adolescents, it should be noted that we had a relatively smaller sample of GM than the other two groups, and the result patterns could be subject to a specific cohort effect. Future prospective studies with a more balanced sample of cisgender heterosexual adolescents and SGMA are needed to confirm the prospective association between sleep and socioemotional outcomes. Still, our results indicated that good sleep quality contributed to healthy socioemotional development among SGMA. From a prevention perspective, most of the risk and protective factors identified for the socioemotional outcomes among these groups are beyond adolescent’s control e.g., socioeconomic status (Chen & Shiu, 2017), legal status regarding same-sex marriage rights (Martin-Storey et al., 2018), or could be quite sensitive topics among SGMA, e.g., family and peer relationship (Patterson et al., 2018). The sleep patterns studied in this paper are instead easily assessed and could be improved in individual/group intervention (Espie, 2009).

### Limitations

The study has several important limitations. For instance, we did not investigate the underlying reason for the disparity in sleep patterns. There were some unexamined, yet important socioemotional outcomes, relevant to adolescent gender development. In particular, the role of minority stress factors, e.g., homophobia in school, or the SGMA outness, use of hormones, and stage of transition among GM are all worth further investigation (Goldhammer et al., 2019; Martin-Storey et al., 2020). Also, we primarily used self-report measures, and future studies using objective measures (e.g., actigraphy) might allow a more comprehensive investigation. Yet, subjective perception of sleep has been consistently found to relate to socioemotional and cognitive outcomes (e.g., Lau et al., 2019). In addition, the measure of SGMA available in MCS did not capture sexual behaviors, but more about self-reported sexual and gender identity. Also, there is a much smaller number of GM than SM and cisgender heterosexual adolescents in this study, which could have affected the results regarding the prevalence of ethnic minority adolescents or the association between sleep and socioemotional outcomes among GM. Still, the prevalence of GM (1.13%) in this study is consistent with the UK 2021 census data (1.08%) (Office for National Statistics, 2021). Future studies with a larger sample could further address the role of GM status, as well as its intersection with other minority factors, e.g., ethnic minority status, in socioemotional outcomes. Moreover, the peer relationship measures had unsatisfactory internal consistency and the participants’ relationships with their mother and father were each measured by 1 item only. Future research might complement our analyses with more comprehensive measures of SGMA status and relationships with peers and parents.

### Conclusion

While existing studies showed disparities in socioemotional outcomes between SGMA and their cisgender heterosexual counterparts, our findings showed that the worst socioemotional outcomes were found among GM, in comparison to SM or cisgender heterosexual adolescents, with the disparity increasing from age 14 to age 17. In addition, we provided novel data suggesting that while better sleep patterns prospectively predicted better socioemotional outcomes, such associations varied across SGMA, with the strongest effects of sleep on socioemotional outcomes among gender minority adolescents. Collectively, further research and clinical attention regarding SGMA, particularly gender minority adolescents’ socioemotional outcomes, are needed, and sleep health might potentially be a target for early identification of SGMA at risk of poor socioemotional outcomes.

### Supplementary Information

Below is the link to the electronic supplementary material.

Supplementary material 1 (DOCX 170.4 kb)

## Data Availability

This is a secondary data analysis study where no new data is generated. The data, material, and code that support the findings of this study are openly available on the Millennium Cohort Study homepage
