# Correction: Sleep and Socioemotional Outcomes Among Sexual and Gender Minority Adolescents: A Longitudinal Study

**DOI:** 10.1007/s10508-024-02885-7

**Published:** 2024-05-13

**Authors:** Mark Lawrence Wong, Jason M. Nagata, Manuela Barreto

**Affiliations:** 1 Department of Social and Behavioural Sciences, City University of Hong Kong, Tat Chee Avenue, Kowloon, Hong Kong; 2 Division of Adolescent & Young Adult Medicine, Department of Pediatrics, University of California, San Francisco, CA USA; 3 Department of Psychology, University of Exeter, Devon, UK

**Correction to: Archives of Sexual Behavior (2023) 53:543–553** 10.1007/s10508-023-02732-1

The article “Sleep and Socioemotional Outcomes Among Sexual and Gender Minority Adolescents: A Longitudinal Study”, written by Mark Lawrence Wong, Jason M. Nagata, and Manuela Barreto, was originally published electronically on the publisher’s internet portal on 22 November 2023 without open access. With the author(s)’ decision to opt for open access, the copyright of the article changed on 25 April 2024 to © The Author(s) 2024 and the article is forthwith distributed under a Creative Commons Attribution 4.0 International License, which permits use, sharing, adaptation, distribution and reproduction in any medium or format, as long as you give appropriate credit to the original author(s) and the source, provide a link to the Creative Commons license, and indicate if changes were made. The images or other third party material in this article are included in the article's Creative Commons license, unless indicated otherwise in a credit line to the material. If material is not included in the article's Creative Commons license and your intended use is not permitted by statutory regulation or exceeds the permitted use, you will need to obtain permission directly from the copyright holder. To view a copy of this license, visit http://creativecommons.org/licenses/by/4.0/.

