# Supplementary material for: Sleep and Socioemotional Outcomes Among Sexual and Gender Minority Adolescents: A Longitudinal Study
Source: Arch Sex Behav. 2023 Nov 22;53(2):543–53. doi: 10.1007/s10508-023-02732-1 (PMC11078824; doi:10.1007/s10508-023-02732-1)
Supplement: Supplementary file 1 — Supplementary material 1 (DOCX 170.4 kb) [file 10508_2023_2732_MOESM1_ESM.docx]

**Supplementary Figure 1**. Significant effects of sleep on socioemotional functions among cisgender heterosexual adolescents


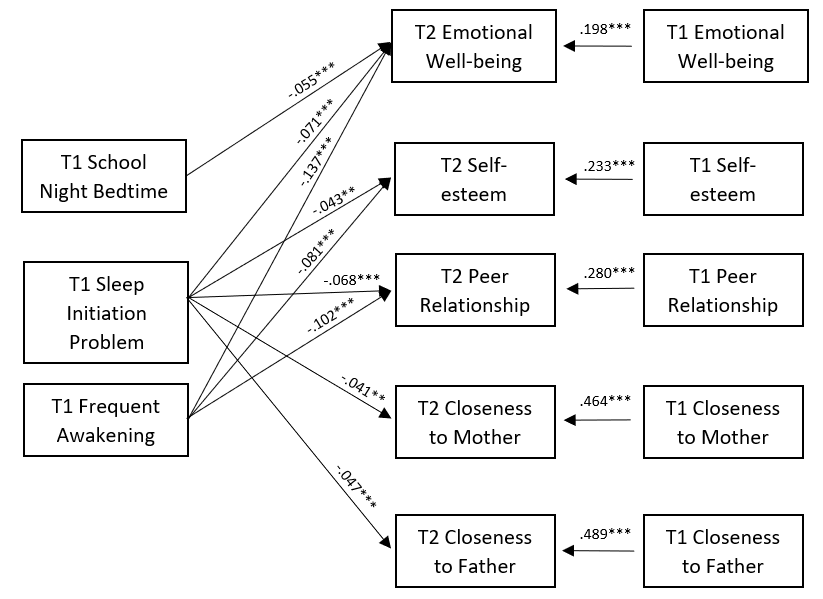


**Supplementary Figure 2**. Significant effects of sleep on socioemotional functions among gender minority adolescents


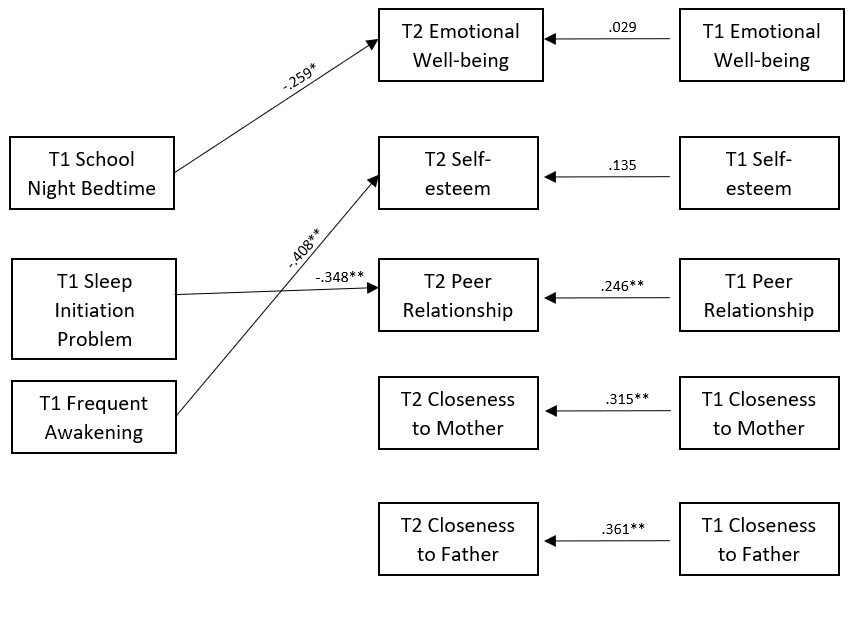


**Supplementary Figure 3**. Significant effects of sleep on socioemotional functions among cisgender sexual minority adolescents


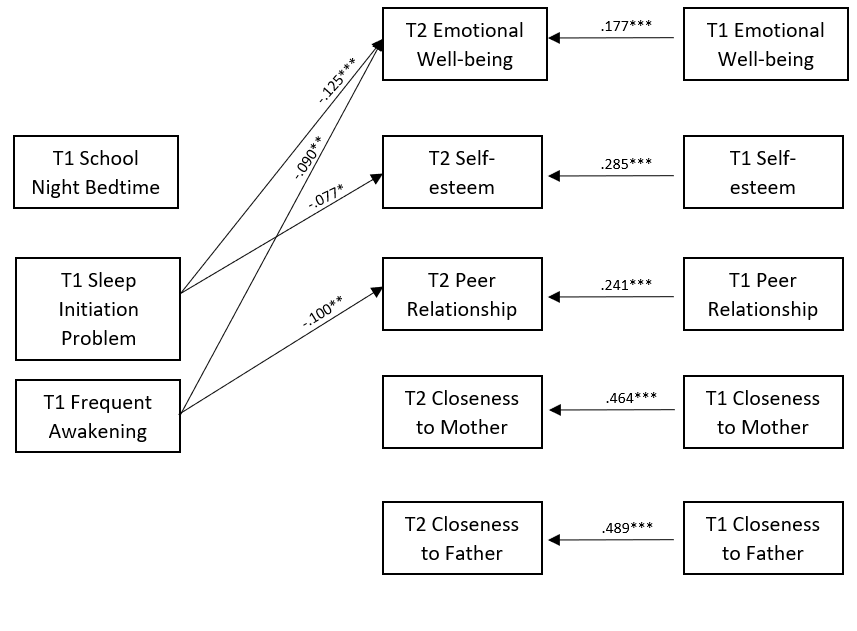


| **Supplementary Table 1**. Effects of demographic factors on socioemotional outcomes among cisgender heterosexual, cisgender sexual minority and gender minority adolescents | | | | | | |
| --- | --- | --- | --- | --- | --- | --- |
|  | Cisgender  Heterosexual | | Cisgender  Sexual Minority | | Gender Minority | |
|  | Est (SE) | Beta | Est (SE) | Beta | Est (SE) | Beta |
| Outcome: **Emotional Well-being** | | | | | | |
| - Age | .014(.07) | .003 | -.075 (.15) | -.014 | -.368 (.70) | -.065 |
| - Sex | -1.242(.06)*** | -.274 | -.990 (.15)*** | -.190 | -1.452 (.74) | -.233 |
| - Family Income | < -.001(.00) | -.019 | <-.001 (.00) | .055 | < -.001 (.00) | .011 |
| - Ethnicity | .461 (.08)*** | .086 | .507 (.21)* | .072 | -.368 (.99) | -.045 |
| Outcome: **Self-esteem** | | | | | | |
| - Age | -.121 (.10) | -.018 | .060 (.20) | .009 | -.653 (1.00) | -.080 |
| - Sex | -.349 (.09)*** | -.058 | -.314 (.20) | -.047 | -1.08 (1.09) | -.120 |
| - Family Income | <.001 (.00) | .030 | < -.001 (.00) | .046 | <-.001 (.00) | .036 |
| - Ethnicity | .237 (.11)* | .033 | -.082 (.27) | -.009 | 1.96 (1.45) | .164 |
| Outcome: **Peer Relationship** | | | | | | |
| - Age | -.084 (.05) | -.024 | .104 (.11) | .027 | -.603 (.53) | -.132 |
| - Sex | -.024 (.05) | -.538 | .111 (.11) | .029 | -.867 (.58) | -.172 |
| - Family Income | <.001 (.00)*** | 5.402 | .001 (.00)*** | .127 | .004 (.00)* | .250 |
| - Ethnicity | .277 (.06)*** | 4.692 | .149 (.15) | .029 | -.120 (.76) | -.018 |
| Outcome: **Relationship with Mother** | | | | | | |
| - Age | .032 (.02) | .019 | .030 (.05) | .017 | -.105 (.24) | -.056 |
| - Sex | .040 (.02)* | .027 | -.009 (.05) | -.005 | -.314 (.26) | -.152 |
| - Family Income | < -.001 (.00) | -.029 | <-.001 (.00) | -.020 | .001 (.00) | .182 |
| - Ethnicity | .021 (.03) | .012 | -.029 (.07) | -.013 | .190 (.35) | .070 |
| Outcome: **Relationship with father** | | | | | | |
| - Age | .070 (.03)** | .037 | .0123 (.05)* | .064 | -.020 (.26) | -.010 |
| - Sex | -.085 (.02)*** | -.049 | -.048 (.05) | -.025 | -.17 (.29) | -.076 |
| - Family Income | < -.001 (.00) | -.022 | <.001 (.00) | <.001 | <-.001 (.00) | .089 |
| - Ethnicity | -.059 (.03)* | -.029 | -.112 (.07) | -.044 | .272 (.38) | .093 |

**Supplementary Table 2.** Intercorrelations among all study variables

|  | 1 | 2 | 3 | 4 | 5 | 6 | 7 | 8 | 9 | 10 | 11 |
| --- | --- | --- | --- | --- | --- | --- | --- | --- | --- | --- | --- |
| 1 School Night Bedtime | - |  |  |  |  |  |  |  |  |  |  |
| 2 Sleep Initiation Problem | .213*** | - |  |  |  |  |  |  |  |  |  |
| 3 Frequent Awakening | -.086*** | -.320*** | - |  |  |  |  |  |  |  |  |
| 4 Emotional Well-being | -.103*** | -.192*** | .234*** | - |  |  |  |  |  |  |  |
| 5 Self-esteem | -.096*** | -.146*** | .175*** | .471*** | - |  |  |  |  |  |  |
| 6 Peer Relationship | -.060*** | -.157*** | .193*** | .376*** | .311*** | - |  |  |  |  |  |
| 7 Closeness to Mother | -.085*** | -.088*** | .072*** | .137*** | .201*** | .154*** | - |  |  |  |  |
| 8 Closeness to Father | -.121*** | -.110*** | .099*** | .216*** | .235*** | .180*** | .598*** | - |  |  |  |
| 9 Age | .075*** | .004 | -.012 | -.008 | <.001 | .015 | .002 | .025 | - |  |  |
| 10 Sex | .009 | .072*** | -.138*** | -.329*** | -.154*** | -.036*** | -.008 | -.112*** | .005 | - |  |
| 11 Family Income | -.076*** | -.005 | .132*** | -.003 | .037*** | .137*** | -.012 | -.014 | .013 | -.006 | - |
| 12 Ethnicity | -.058*** | -.031*** | -.037*** | .089*** | .056*** | .024* | .042* | -.028* | -.023* | -.005 | -.279*** |

**p* < .05*, ***p* < .001

**Supplementary Results**

**Differences Between Groups in Socioemotional Outcomes**

For self-esteem, at age 14, *F* (2, 8551) = 128.855, $\eta_{p}^{2}$ = .029, *p* < .001, the SM and GM groups had significantly lower self-esteem than the cisgender heterosexual group, *ps*_bonferonni_ < .001. At age 17, *F* (2, 8718) = 184.421, $\eta_{p}^{2}$ = .041, *p* < .001, the GM group had the lowest self-esteem score, followed by the SM and then cisgender heterosexual group *ps*_bonferonni_ < .001. Similarly, for emotional well-being at age 14: *F* (2, 8518) = 39.054, $\eta_{p}^{2}$ = .009, *p* < .001, and age 17: *F* (2, 8620) = 344.827, $\eta_{p}^{2}$ = .074, *p* < .001, and peer relationships at age 14: *F* (2, 8518) = 216.660, $\eta_{p}^{2}$ = .017, *p* < .001, and age 17: *F* (2, 8620) = 180.203, $\eta_{p}^{2}$ = .040, *p* < .001. That is, GM participants had the lowest level of emotional well-being and peer relationships, followed by the SM, and then cisgender heterosexual group, *ps*_bonferroni_ ≤ .001. Regarding their relationships with their mother, at age 14, *F* (2, 8620) = 180.203, $\eta_{p}^{2}$ = .040, *p* < .001, SM participants reported significantly worse relationships with their mother than cisgender heterosexual adolescents, *p*_bonferroni_ = .021. At age 17, *F* (2, 8240) = 42.171, $\eta_{p}^{2}$ = .010, *p* < .001, both GM and SM participants reported significantly worse relationships with their mother when compared to cisgender heterosexual adolescents, *ps*_bonferonni_ ≤ .001. For relationships with their father, at age 14, *F* (2, 8658) = 14.581, $\eta_{p}^{2}$ = .003, *p* < .001, SM participants reported significantly worse relationships with their father than cisgender heterosexual adolescents, *p*_bonferonni_ < .001. At age 17, *F* (2, 5895) = 56.226, $\eta_{p}^{2}$ = .019, *p* < .001, GM participants reported significantly worse relationships with their father when compared to the SM, *p*_bonferonni_ = .023, and the cisgender heterosexual group, *p*_bonferonni_ < .001 (**Table 2**).

**Prospective Association Between Sleep and Socioemotional Outcomes**

Among cisgender heterosexual adolescents, earlier bedtime was found to prospectively predict better emotional well-being, beta = -.055, *p* < .001. Less severe sleep initiation problems predicted all of the socioemotional outcomes, *p*s < .01, and less frequent awakening prospectively predicted better emotional well-being, beta = -.137, *p* < .001, higher self-esteem, beta = -.081, *p* < .001, and better peer relationships, beta = -.103, *p* < .001 (**Supplementary Figure 1**).

Among SM, less severe sleep initiation problems predicted better emotional well-being, beta = -.125, *p* < .001, and higher self-esteem, beta = -.077, *p* = .016; while less frequent awakening predicted better emotional well-being, beta = -.090, *p* = .005, and better peer relationships, beta = -.097, *p* = .002. The sleep variables were not found to predict relationship with parents, *p*s > .05 (**Supplementary Figure 2**).

Among GM, earlier bedtime significantly predicted better emotional well-being, beta = -.249, *p* = .041, less severe sleep initiation problem predicted better peer relationships, beta = -.348, *p* = .008, and less frequent nocturnal awakening predicted higher self-esteem, beta = -.408, *p* = .002, though none of the sleep variables significantly predicted relationship with parents, *p*s > .05 (**Supplementary Figure 3**).
